# Supplementary material for: Reaction Pathway Analysis of Methane and Propylene Cracking: A Reactive Force Field Simulation Approach
Source: Materials (Basel). 2025 Jun 6;18(12):2672. doi: 10.3390/ma18122672 (PMC12194203; doi:10.3390/ma18122672)
Supplement: Supplementary file 1 [file materials-18-02672-s001.zip › materials-3616558-supplementary.pdf]

Reaction Pathway Analysis of Methane and Propylene Cracking: A Reactive Force Field Simulation Approach

Wei Yang[a], Hongyang Cui[a], Geng Chen[a], Yiqiang Hong[a*], Zhen Dai[a], Dabo Xing[a], Qiaosheng Li[a], Yunlong Ma[a], Lei liang[a], Hongyang Cui[a] and Youpei Du[a*]

a Beijing System Design Institute of Mechanical-Electrical Engineering, Beijing, 100871, China

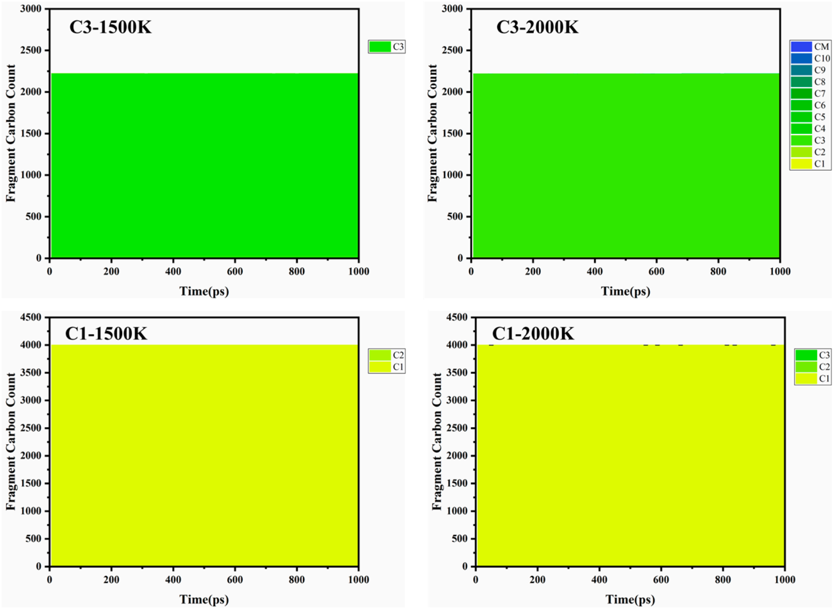

Figure S1 Product monitoring and statistical analysis: Statistics of products of propylene and methane at different temperatures, where CM refers to carbon fragments with more than 10 carbon atoms

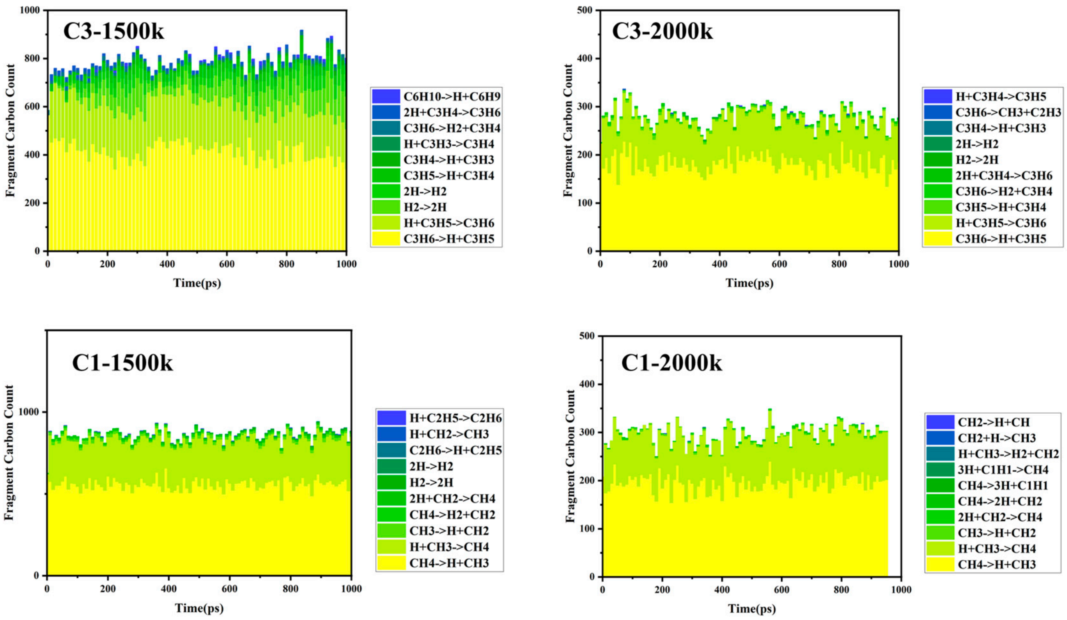

Figure S2 Comprehensive Reaction Pathway Identification and Statistical Analysis of All Methane Species
